# Influenza A(H9N2) Virus, Burkina Faso

**DOI:** 10.3201/eid2312.171294

**Published:** 2017-12

**Authors:** Bianca Zecchin, Germaine Minoungou, Alice Fusaro, Sidi Moctar, Anne Ouedraogo-Kaboré, Alessia Schivo, Annalisa Salviato, Sabrina Marciano, Isabella Monne

**Affiliations:** Istituto Zooprofilattico Sperimentale delle Venezie, Legnaro, Padova, Italy (B. Zecchin, A. Fusaro, A. Schivo, A. Salviato, S. Marciano, I. Monne);; Laboratoire National d’Elevage de Ouagadougou, Ouagadougou, Burkina Faso (G. Minoungou, S. Moctar, A. Ouedraogo-Kaboré)

**Keywords:** influenza, influenza virus, viruses, H9N2 subtype, phylogeny, respiratory infections, poultry, zoonoses, Burkina Faso, West Africa

## Abstract

We identified influenza A(H9N2) virus G1 lineage in poultry in Burkina Faso. Urgent actions are needed to raise awareness about the risk associated with spread of this zoonotic virus subtype in the area and to construct a strategy for effective prevention and control of influenza caused by this virus.

Since their detection in China in 1992, influenza A(H9N2) viruses have caused large economic losses to the poultry industry and have occasionally been transmitted to mammalian species, including humans. Three main genetic lineages were described among the Eurasian H9N2 subtype viruses: G1, Y280, and Y439 (Korean) lineage (*1*). In the past decade, the G1 lineage has spread mostly in gallinaceous birds across Asia, the Middle East, and eventually North Africa, where H9N2 outbreaks were reported in Libya (2006 and 2013) (*2*), Tunisia (2010–2012) (*3*), Egypt (2011–present), and Morocco (2016) (*4*).

The Veterinary Services of Ouagadougou, Burkina Faso, submitted 30 tracheal swab specimens and 10 organ samples collected in January 2017 in Burkina Faso to the World Organisation for Animal Health/Food and Agriculture Organization of the United Nations Reference Laboratory for Avian Influenza, Istituto Zooprofilattico Sperimentale delle Venezie (Legnaro, Padova, Italy). All samples were collected from a layer farm that was experiencing decreased egg production and respiratory signs among its flock; the animals were suspected to have infectious bronchitis virus (IBV).

Molecular analyses of the animal samples showed negative results for IBV and indicated that animals from the farm were infected with avian influenza A(H9N2) virus. The 8 gene segments were obtained for 1 representative virus by using a MiSeq Platform (Illumina, San Diego, CA, USA). Sequences were submitted to GenBank under accession numbers MF510849–56.

The maximum-likelihood phylogenetic tree of the hemagglutinin (HA) gene showed that the H9N2 subtype virus from Burkina Faso belonged to the G1 lineage, which has remarkable zoonotic potential. This virus clustered with H9N2 subtype viruses isolated in Morocco in 2016 (99.2% similarity) and with an H9N2 subtype virus identified in the United Arab Emirates in 2015 (A/chicken/Dubai/D2506.A/2015) (98.7% similarity) (Technical Appendix Figure 1). Phylogenetic trees obtained for all other gene segments confirmed clustering with viruses from Morocco and the United Arab Emirates, similar to that observed for HA gene phylogeny.

Phylogeographic analysis (online Technical Appendix) identified multiple introductions of influenza A(H9N2) virus into North Africa from the Middle East and Pakistan. The H9N2 subtype virus identified in Burkina Faso seems to have originated from Morocco, although we cannot rule out the possibility that H9N2 subtype viruses were circulating in unsampled locations (Technical AppendixFigure 2 and Video).

**Video:**
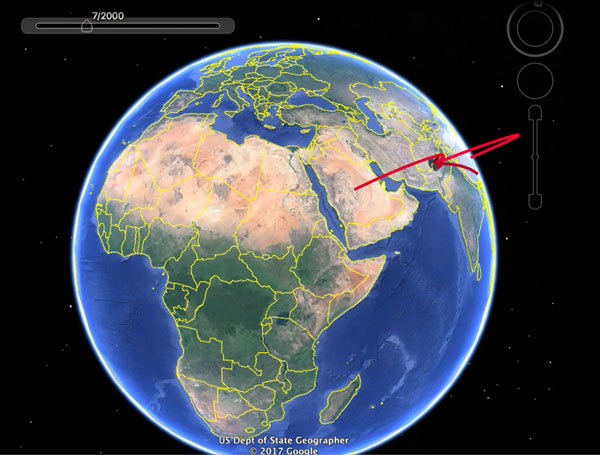
Spread of influenza A(H9N2) virus over time from the United Arab Emirates to Morocco and Burkina Faso. Animation shows the phylogeographic reconstruction resulting from the maximum clade credibility phylogenetic tree obtained in SPREAD version 1.0.6 (https://github.com/phylogeography/SPREAD/issues/7) (video forthcoming).

Analyses of the deep sequencing data showed that ≈50% of the virus population in the tracheal swab specimen had leucine at position 226 (H3 numbering) of the HA receptor binding site (sequence coverage of 14,152 reads in the indicated position), which enables preferential binding to human-like α2–6-linked sialic acid receptors (*5*). Furthermore, a potential additional glycosylation site (NLS), which had not previously been detected in the G1 lineage, was identified at positions 271–273 (H3 numbering). In the acidic polymerase protein, the H9N2 subtype virus from Burkina Faso had the mutation PA-S409N, which is considered a host specificity marker of human influenza virus (*6*). The same mutation was detected in related viruses from Morocco and Dubai.

Identification of H9N2 subtype virus in West Africa, where highly pathogenic H5 strains of the A/goose/Guangdong/1/1996 lineage (Gs/GD) have been widely circulating since the beginning of 2015, is a concern because of animal health implications, negative effects on local economies, and possible emergence of reassortant viruses with unknown biological properties. Reassortment events between H9N2 and highly pathogenic H5N1 subtype viruses were reported in China in 2005 and 2016 (*7**,**8*) and in Bangladesh in 2012 (*9*). In December 2013, an H5N1 subtype virus that had an H9N2 subtype polymerase basic 2 gene was reported in a patient in Canada who had returned from China (*10*). Moreover, H5N6 subtype reassortant viruses belonging to clade 2.3.4.4, which contain H9N2 subtype–like internal genes, were identified in China in 2015–2016 (*8*).

H5 strains belonging to clades 2.3.2.1c and 2.3.4.4 are currently circulating in West Africa. This finding, combined with detection of human-like receptor specificity and 2 mutations typical of human influenza viruses in the H9N2 subtype virus from Burkina Faso, might indicate emergence of a strain capable of infecting humans and warrants additional attention to the avian influenza situation in West Africa. Furthermore, identification of H9N2 subtype viruses in Morocco and Burkina Faso in chickens suggests that commercial poultry trade between North and West Africa might have played a key role in spread of the virus.

Involvement of wild birds in long-distance spread of H9N2 subtype G1 virus seems unlikely because this lineage is strongly adapted to poultry. These observations highlight the difficulty in tracing and containing circulating H9N2 subtype G1 virus and underline the need to review current approaches of disease reporting to understand spread and effects of this virus, which are probably underestimated. Thus, it is imperative to provide strategic guidance to countries in West Africa on technical and policy options for cost-effective surveillance and prevention and control of multiple cocirculating influenza virus strains.

Technical Appendix Additional information on influenza A(H9N2) virus, Burkina Faso.
