# Supplementary material for: Influenza A(H9N2) Virus, Burkina Faso
Source: Emerg Infect Dis. 2017 Dec;23(12):2118–9. doi: 10.3201/eid2312.171294 (PMC5708222; doi:10.3201/eid2312.171294)
Supplement: Technical Appendix — Additional information on influenza A(H9N2) virus, Burkina Faso. [file 17-1294-Techapp-s1.pdf]

Article DOI: https://doi.org/10.3201/eid2312.171294

Influenza A(H9N2) Virus, Burkina Faso

Technical Appendix

Materials and Methods

Genome Amplification and Sequencing

We purified influenza virus RNA from clinical samples by using the Nucleospin RNA Kit (Macherey–Nagel, Duren, Germany). We amplified the complete genome of A/chicken/Burkina_Faso/17RS93–19/2017(H9N2) virus by using the SuperScript III One-Step RT-PCR System and Platinum Taq High Fidelity (Invitrogen, Carlsbad, CA, USA) as described (*1*). The sequencing library was prepared by using the Nextera DNA XT Sample preparation kit (Illumina, San Diego, CA, USA) and quantified by using the Qubit dsDNA High Sensitivity Kit (Invitrogen, Carlsbad, CA, USA). The High Sensitivity DNA Analysis Kit (Agilent Technologies, Alpharetta, GA, USA) was used to determine average fragment length. According to the manufacturer's instructions, the library was sequenced by using Illumina MiSeq (2 × 250-bp paired-end).

Illumina Sequencing Data Analysis

FastQC version 0.11.2 (https://www.bioinformatics.babraham.ac.uk/projects/fastqc/) was used to assess read quality. Raw data were filtered by removal of reads with >10% of undetermined bases, reads with >100 bases with a Q score <7, and duplicated paired-end reads. Remaining reads were clipped from Nextera XT adaptors (Illumina) with scythe version 0.991 (https://github.com/vsbuffalo/scythe) and trimmed with sickle version 1.33 (https://github.com/najoshi/sickle). High-quality reads $\geq$80 bases were aligned against a reference genome by using BWA version 0.7.12 (*2*). Picard-tools version 2.1.0 (http://picard.sourceforge.net) and GATK version 3.5 (*3*–*5*) were used to correct potential errors, realign reads around indels, and recalibrate base quality. LoFreq version 2.1.2 (*6*) was used to call single-nucleotide polymorphisms. Outputs were used to generate consensus sequences.

Phylogenetic Analyses

Consensus sequences of each gene segment of A/chicken/Burkina_Faso/17RS93–19/2017(H9N2) virus were compared with the most related sequences available in GISAID

(https://www.gisaid.org/) and aligned by using MAFFT version 7 (*7*). Maximum-likelihood phylogenetic trees were obtained by using the best-fit general time-reversible model of nucleotide substitution with gamma-distributed rate variation among sites (with 4 rate categories, Γ4) and a heuristic subtree pruning and regrafting branch-swapping search (*8*) implemented in PhyML version 3.1 (http://www.atgc-montpellier.fr/phyml/versions.php). Bootstrap analysis with 100 replicates was performed for each tree to assess support for nodes. Phylogenetic trees were visualized by using FigTree version 1.4.2 (http://tree.bio.ed.ac.uk/software/figtree/).

Bayesian Analysis

A time-scaled Bayesian analysis of the hemagglutinin gene was performed by using the Markov chain Monte Carlo method available in BEAST version 1.8.4 (http://beast.community/2016-06-17_BEAST_v1.8.4_released.html). A Hasegawa-Kishino-Yano 85 + Γ_4 model of nucleotide substitution with 2 data partitions of codon positions (1st and 2nd positions, 3rd position) was used, and base frequencies were unlinked across all codon positions (SRD06 substitution model). We used a relaxed uncorrelated lognormal molecular clock and a Skyride coalescent model in BEAST. Chain lengths were run for 50 million iterations to achieve convergence as assessed by using Tracer version 1.6 (http://beast.bio.ed.ac.uk/Tracer). TreeAnnotator version 1.8.4 (*9*) was used to generate the maximum clade credibility (MCC) phylogenetic tree, and we adopted an appropriate burn-in (10% of trees). The MCC tree was visualized by using FigTree version 1.4.2 (http://tree.bio.ed.ac.uk/software/figtree/). SPREAD version 1.0.6 (https://www.kuleuven.be/aidslab/phylogeography/SPREAD.html) (*10*) was used to visualize the phylogeographic reconstruction resulting from the MCC phylogenetic tree and to identify the well-supported rates, calculating the Bayes factors. An animation of viral spread over time is shown in the video (https://wwwnc.cdc.gov/EID/article/23/12/17-1294-V1.htm).

References

1. Zhou B, Donnelly ME, Scholes DT, St George K, Hatta M, Kawaoka Y, et al. Single-reaction genomic amplification accelerates sequencing and vaccine production for classical and Swine origin human influenza a viruses. J Virol. 2009;83:10309–13. PubMed http://dx.doi.org/10.1128/JVI.01109-09

2. Li H, Durbin R. Fast and accurate long-read alignment with Burrows-Wheeler transform. Bioinformatics. 2010;26:589–95. PubMed http://dx.doi.org/10.1093/bioinformatics/btp698

3. McKenna A, Hanna M, Banks E, Sivachenko A, Cibulskis K, Kernytsky A, et al. The Genome Analysis Toolkit: a MapReduce framework for analyzing next-generation DNA sequencing data. Genome Res. 2010;20:1297–303. PubMed http://dx.doi.org/10.1101/gr.107524.110

4. DePristo MA, Banks E, Poplin R, Garimella KV, Maguire JR, Hartl C, et al. A framework for variation discovery and genotyping using next-generation DNA sequencing data. Nat Genet. 2011;43:491–8. PubMed http://dx.doi.org/10.1038/ng.806

5. Van der Auwera GA, Carneiro MO, Hartl C, Poplin R, Del Angel G, Levy-Moonshine A, et al. From FastQ data to high confidence variant calls: the Genome Analysis Toolkit best practices pipeline. Curr Protoc Bioinformatics. 2013;43:11.10.1–33. doi: 10.1002/0471250953.bi1110s43.

6. Wilm A, Aw PP, Bertrand D, Yeo GH, Ong SH, Wong CH, et al. LoFreq: a sequence-quality aware, ultra-sensitive variant caller for uncovering cell-population heterogeneity from high-throughput sequencing datasets. Nucleic Acids Res. 2012;40:11189–201. PubMed http://dx.doi.org/10.1093/nar/gks918

7. Katoh K, Standley DM. MAFFT multiple sequence alignment software version 7: improvements in performance and usability. Mol Biol Evol. 2013;30:772–80. PubMed http://dx.doi.org/10.1093/molbev/mst010

8. Guindon S, Gascuel O, Rannala B. A simple, fast, and accurate algorithm to estimate large phylogenies by maximum likelihood. Syst Biol. 2003;52:696–704. PubMed http://dx.doi.org/10.1080/10635150390235520

9. Drummond AJ, Rambaut A. BEAST: Bayesian evolutionary analysis by sampling trees. BMC Evol Biol. 2007;7:214. PubMed http://dx.doi.org/10.1186/1471-2148-7-214

10. Bielejec F, Rambaut A, Suchard MA, Lemey P. SPREAD: spatial phylogenetic reconstruction of evolutionary dynamics. Bioinformatics. 2011;27:2910–2. PubMed http://dx.doi.org/10.1093/bioinformatics/btr481

Technical Appendix Table 1. Bayes factor test results for significant nonzero rates of influenza A(H9N2) viruses

Pairs of locations with Bayes factor >5	Bayes factor
Pakistan-Afghanistan and South Asia	3,795.26
Iran-Iraq and Pakistan-Afghanistan	1,603.01
Saudi Arabia-Qatar and United Arab Emirates	1,436.70
Egypt and Israel-Jordan-Lebanon	658.83
Burkina Faso and Morocco	112.11
Morocco and United Arab Emirates	58.59
Libya and Saudi Arabia-Qatar	22.48
Pakistan-Afghanistan and United Arab Emirates	13.25
Israel-Jordan-Lebanon and United Arab Emirates	13.23
Pakistan-Afghanistan and Tunisia	7.43

Technical Appendix Table 2. Hemagglutinin gene segments of 16 influenza virus strains used for analysis of influenza A(H9N2) virus, Burkina Faso*

Segment ID	Country	Collection date	Isolate name	Originating laboratory	Submitting laboratory	Authors†
EPI457491	Bangladesh	2009 Mar 5	A/duck/Bangladesh/1009/2009	Institute of Epidemiology Disease Control and Research and Bangladesh National Influenza Centre	Centers for Disease Control and Prevention	NA
EPI557489	Egypt	2013 Feb 14	A/chicken/Egypt/NLQP123VD-AR758/2013		Friedrich-Loeffler-Institut	Naguib MM, Arafa AM, Selim AA, Hassan MK, Beer M, Harder TC
EPI355122	Egypt	2011 Mar 5	A/chicken/Egypt/11vir4453–280/2011	Istituto Zooprofilattico Sperimentale Delle Venezie	Istituto Zooprofilattico Sperimentale Delle Venezie	Monne I, Hussein HA, Fusaro A, Valastro V, Hamoud MM, Rabab A, Noseir S, Capua I, Cattoli G
EPI355114	Egypt	2010 Dec 9	A/chicken/Egypt/11vir4453–276/2010	Istituto Zooprofilattico Sperimentale Delle Venezie	Istituto Zooprofilattico Sperimentale Delle Venezie	Monne I, Hussein HA, Fusaro A, Valastro V, Hamoud MM, Rabab A, Noseir S, Capua I, Cattoli G
EPI355106	Egypt	2011 Mar 5	A/chicken/Egypt/11vir4453–275/2011	Istituto Zooprofilattico Sperimentale Delle Venezie	Istituto Zooprofilattico Sperimentale Delle Venezie	Monne I, Hussein HA, Fusaro A, Valastro V, Hamoud MM, Rabab A, Noseir S, Capua I, Cattoli G
EPI301655	Qatar	2008 Jan 1	A/chicken/Qatar/4576–4/2008	Istituto Zooprofilattico Sperimentale Delle Venezie	Istituto Zooprofilattico Sperimentale Delle Venezie	Fusaro A, Monne I, Salviato A, Valastro V, Schivo A, Amarin NM, Gonzalez C, Ismail MM, Al Blowi MH, Khan OA, Maken Ali AS, Hedayati A, Garcia JG, Ziay GM, Shoushtari A, Al Qahtani KN, Capua I, Holmes EC, Cattoli G
EPI301631	Iran	2009 Jan 1	A/chicken/Iran/10VIR854–4/2009	Istituto Zooprofilattico Sperimentale Delle Venezie	Istituto Zooprofilattico Sperimentale Delle Venezie	Fusaro A, Monne I, Salviato A, Valastro V, Schivo A, Amarin NM, Gonzalez C, Ismail MM, Al Blowi MH, Khan OA, Maken Ali AS, Hedayati A, Garcia JG, Ziay GM, Shoushtari A, Al Qahtani KN, Capua I, Holmes EC, Cattoli G
EPI301615	Iran	2009 Jan 1	A/chicken/Iran/10VIR854–3/2009	Istituto Zooprofilattico Sperimentale Delle Venezie	Istituto Zooprofilattico Sperimentale Delle Venezie	Fusaro A, Monne I, Salviato A, Valastro V, Schivo A, Amarin NM, Gonzalez C, Ismail MM, Al Blowi MH, Khan OA, Maken Ali AS, Hedayati A, Garcia JG, Ziay GM, Shoushtari A, Al Qahtani KN, Capua I, Holmes EC, Cattoli G
EPI301607	Iran	2008 Jan 1	A/chicken/Iran/10VIR854–5/2008	Istituto Zooprofilattico Sperimentale Delle Venezie	Istituto Zooprofilattico Sperimentale Delle Venezie	Fusaro A, Monne I, Salviato A, Valastro V, Schivo A, Amarin NM, Gonzalez C, Ismail MM, Al Blowi MH, Khan OA, Maken Ali AS, Hedayati A, Garcia JG, Ziay GM, Shoushtari A, Al Qahtani KN, Capua I, Holmes EC, Cattoli G
EPI301591	United Arab Emirates	2008-Jan-01	A/chicken/Dubai/09vir3771–2/2008	Istituto Zooprofilattico Sperimentale Delle Venezie	Istituto Zooprofilattico Sperimentale Delle Venezie	Fusaro A, Monne I, Salviato A, Valastro V, Schivo A, Amarin NM, Gonzalez C, Ismail MM, Al Blowi MH, Khan OA, Maken Ali AS, Hedayati A, Garcia JG, Ziay GM, Shoushtari A, Al Qahtani KN, Capua I, Holmes EC, Cattoli G

Segment ID	Country	Collection date	Isolate name	Originating laboratory	Submitting laboratory	Authors†
EPI301498	Jordan	2010 Jan 1	A/chicken/Jordan/436–2/2010	Istituto Zooprofilattico Sperimentale Delle Venezie	Istituto Zooprofilattico Sperimentale Delle Venezie	Fusaro A, Monne I, Salviato A, Valastro V, Schivo A, Amarin NM, Gonzalez C, Ismail MM, Al Blowi MH, Khan OA, Maken Ali AS, Hedayati A, Garcia JG, Ziay GM, Shoushtari A, Al Qahtani KN
EPI301490	Jordan	2010 Jan 1	A/chicken/Jordan/436–1/2010	Istituto Zooprofilattico Sperimentale Delle Venezie	Istituto Zooprofilattico Sperimentale Delle Venezie	Fusaro A, Monne I, Salviato A, Valastro V, Schivo A, Amarin NM, Gonzalez C, Ismail MM, Al Blowi MH, Khan OA, Maken Ali AS, Hedayati A, Garcia JG, Ziay GM, Shoushtari A, Al Qahtani KN, Capua I, Holmes EC, Cattoli G
EPI355392	Egypt	2011 Jan 1	A/chicken/Egypt/11vir4453–132/VRLCU/2011	NA	Istituto Zooprofilattico Sperimentale Delle Venezie	NA
EPI355384	Egypt	2011 Mar 5	A/chicken/Egypt/11vir4453–274/2011	NA	Istituto Zooprofilattico Sperimentale Delle Venezie	NA
EPI223115	Afghanistan	2009 Jan 1	A/chicken/Afghanistan/329–7vir09-AFG-Heart6/2009	NA	Istituto Zooprofilattico Sperimentale Delle Venezie	Valastro V, Salviato A, Fusaro A, Monne I, Habib M, Ziay G, Garcia J, Cattoli G, Capua I

*ID, identification; NA, not available.
†Authors who submitted data may be contacted directly via the GISAID website (https://www.gisaid.org/).

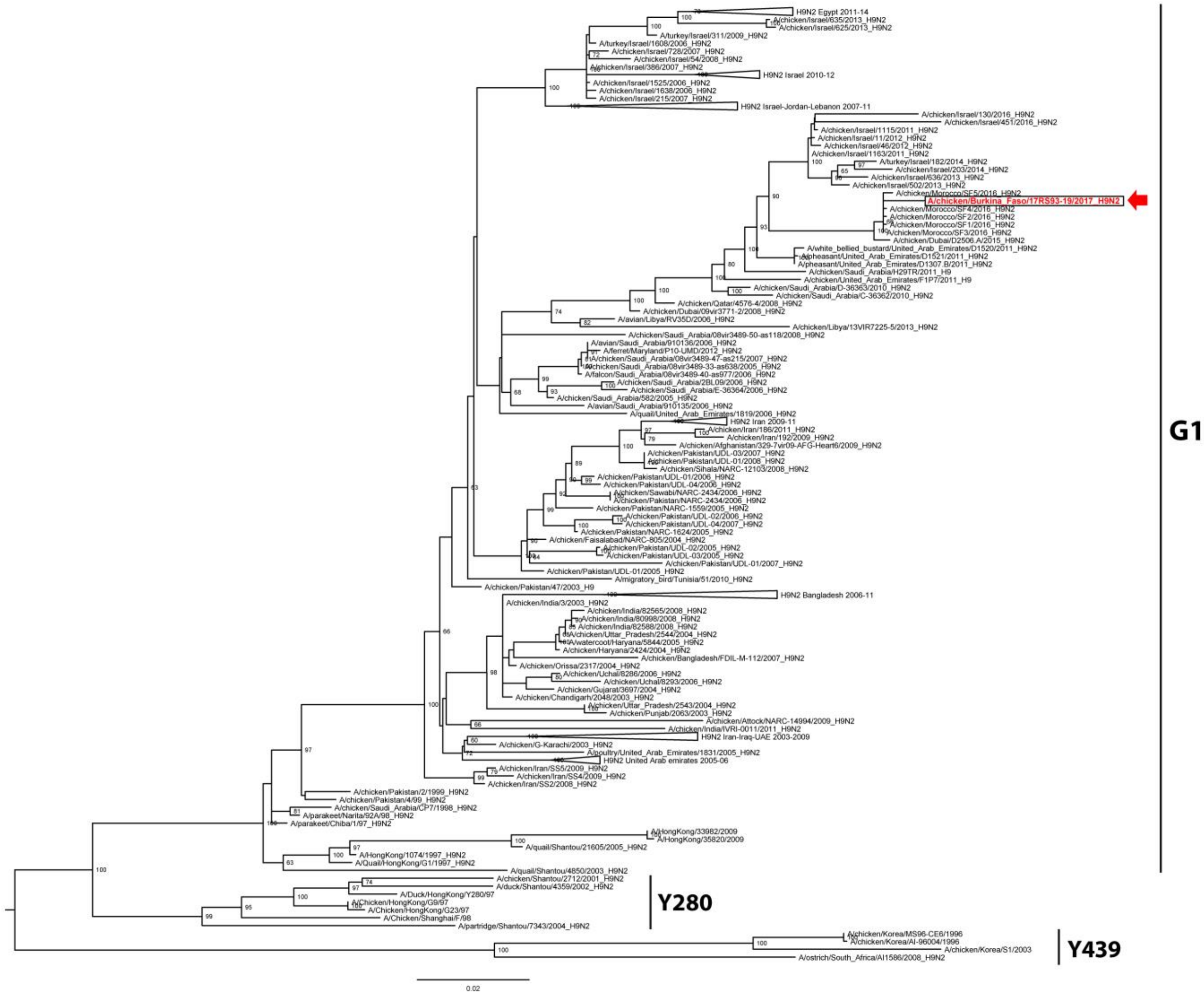

Technical Appendix Figure 1. Maximum-likelihood phylogenetic tree of the hemagglutinin gene of influenza A(H9N2) viruses. Influenza A(H9N2) virus from Burkina Faso is indicated in in red. Bootstrap values >60% are indicated next to nodes. Scale bar indicates nucleotide substitutions per site.

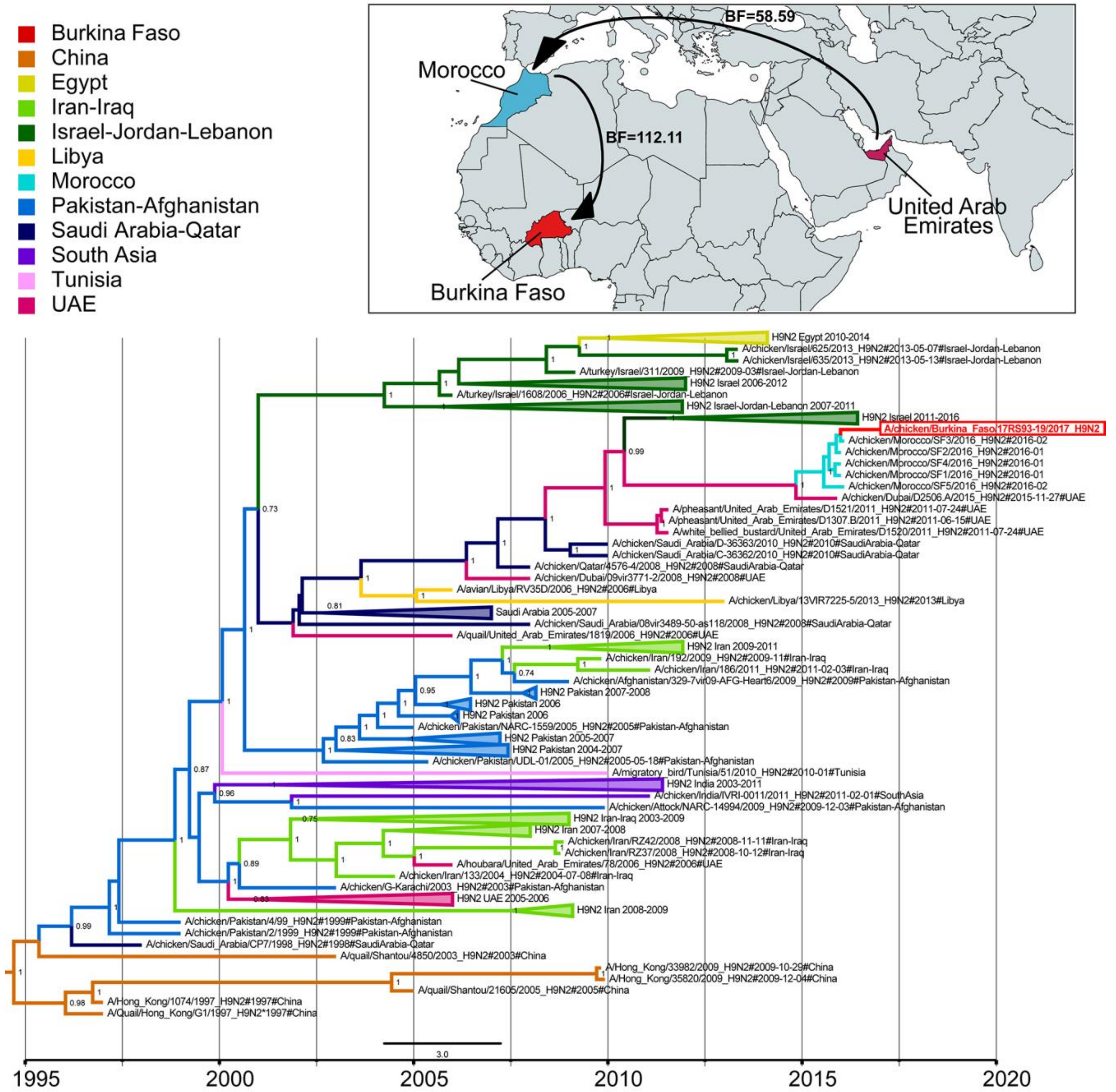

Technical Appendix Figure 2. Maximum clade credibility tree showing evolutionary relationships between A/chicken/Burkina Faso/17RS93–19/2017(H9N2) influenza virus (indicated in red) and influenza A(H9N2) viruses isolated in North Africa, the Middle East, and Asia. Posterior probabilities >70 are provided for each node. Color of each branch indicates location where analyzed viruses were collected. Scale bar indicates nucleotide substitutions per site. Map indicates spread of virus from the United Arab Emirates to Morocco and from Morocco to Burkina Faso. Bayes factors (BF) for significant nonzero rates are indicated next to corresponding arrows. UAE, United Arab Emirates.

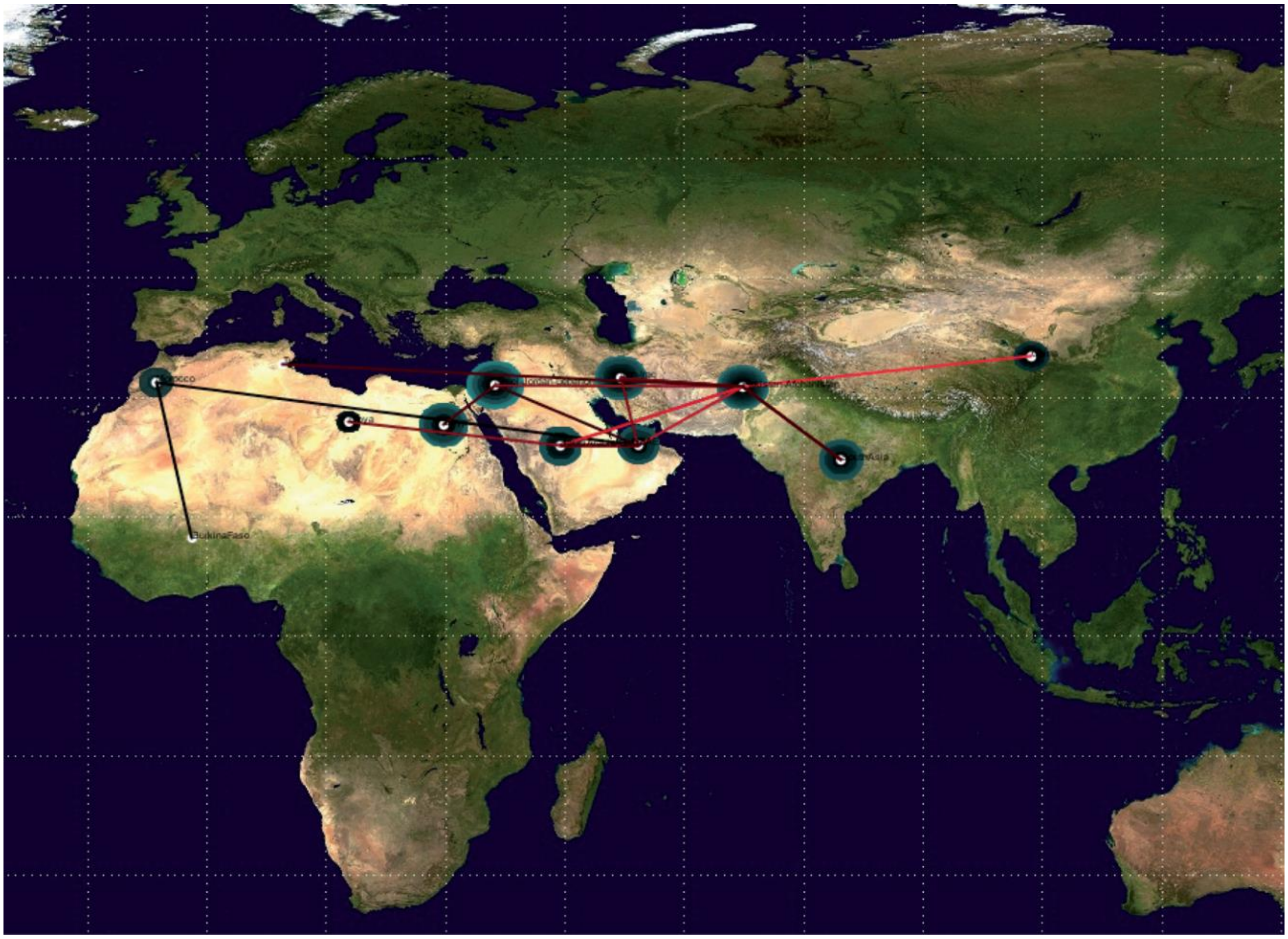

Technical Appendix Figure 3. Spread of influenza A(H9N2) virus in Africa and Asia. Phylogeographic reconstruction resulting from the maximum clade credibility phylogenetic tree obtained with SPREAD version 1.0.6 (https://github.com/phylogeography/SPREAD/issues/7).
